# Genome-Wide Association Identifies a Common Variant in the Reelin Gene That Increases the Risk of Schizophrenia Only in Women

**DOI:** 10.1371/journal.pgen.0040028

**Published:** 2008-02-15

**Authors:** Sagiv Shifman, Martina Johannesson, Michal Bronstein, Sam X Chen, David A Collier, Nicholas J Craddock, Kenneth S Kendler, Tao Li, Michael O'Donovan, F. Anthony O'Neill, Michael J Owen, Dermot Walsh, Daniel R Weinberger, Cuie Sun, Jonathan Flint, Ariel Darvasi

**Affiliations:** 1 Wellcome Trust Centre for Human Genetics, University of Oxford, Oxford, United Kindgdom; 2 Department of Genetics, The Institute of Life Sciences, The Hebrew University of Jerusalem, Jerusalem, Israel; 3 Department of Psychiatry, Virginia Commonwealth University, Richmond, Virginia, United States of America; 4 Virginia Institute for Psychiatric and Behavior Genetics, Virginia Commonwealth University, Richmond, Virginia, United States of America; 5 Social, Genetic and Developmental Psychiatry Centre, Institute of Psychiatry, King's College London, London, United Kingdom; 6 Department of Psychological Medicine, School of Medicine, Cardiff University, Cardiff, United Kingdom; 7 Psychiatric Laboratory and Department of Psychiatry, West China Hospital, Sichuan University, The People's Republic of China; 8 State Key Laboratory of Biotherapy, West China Hospital, Sichuan University, The People's Republic of China; 9 The Department of Psychiatry, The Queens University, Belfast, Northern Ireland; 10 The Health Research Board, Dublin, Ireland; 11 Clinical Brain Disorders Branch, Genes, Cognition, and Psychosis Program, Intramural Research Program, National Institute of Mental Health, National Institutes of Health (NIH), United States Department of Health and Human Services, Bethesda, Maryland, United States of America; North Carolina State University, United States of America

## Abstract

Sex differences in schizophrenia are well known, but their genetic basis has not been identified. We performed a genome-wide association scan for schizophrenia in an Ashkenazi Jewish population using DNA pooling. We found a female-specific association with rs7341475, a SNP in the fourth intron of the reelin (RELN) gene (*p* = 2.9 × 10^−5^ in women), with a significant gene-sex effect (*p* = 1.8 × 10^−4^). We studied rs7341475 in four additional populations, totaling 2,274 cases and 4,401 controls. A significant effect was observed only in women, replicating the initial result (*p* = 2.1 × 10^−3^ in women; *p* = 4.2 × 10^−3^ for gene-sex interaction). Based on all populations the estimated relative risk of women carrying the common genotype is 1.58 (*p* = 8.8 × 10^−7^; *p* = 1.6 × 10^−5^ for gene-sex interaction). The female-specific association between RELN and schizophrenia is one of the few examples of a replicated sex-specific genetic association in any disease.

## Author Summary

Schizophrenia is a complex mental disease, which includes symptoms of delusions, hallucinations, disorganized speech, aberrant behavior, lack of emotional expression, diminished motivation, and social withdrawal. The cause of schizophrenia is unknown, but there is extensive evidence that genetics play a significant role in its aetiology. We studied the genetic basis of schizophrenia by analyzing around 500,000 genetic variants distributed across the whole human genome in DNA from schizophrenic patients and controls. We analyzed separately the DNA from men and women, and identified a genetic variant that increases the risk of developing schizophrenia in women only. The genetic variant is estimated to increase the risk of schizophrenia for women carrying the risk variant by 1.4-fold. The genetic variant is in a gene called reelin, which is known to play a part in brain development. However, it is still unclear how this genetic variant predisposes to schizophrenia nor why it is specific to women only.

## Introduction

Schizophrenia (181500) is a common psychiatric disorder of unknown aetiology. Individual twin studies and meta-analyses of twin studies [1] estimate that the heritability of schizophrenia is approximately 80%. Analysis of family, adoption and twin data indicate that inheritance acts in a complex fashion, in combination with the environment, to mediate the risk of developing the illness [2]. However, despite the relatively large heritability of schizophrenia, efforts to identify the molecular risk factors have so far yielded equivocal results (reviewed in [3]).

Sex differences in the risk of a disorder can provide clues about its pathogenesis. For schizophrenia, the age of onset, premorbid functioning, symptomatic characteristics, and course of illness differ significantly between men and women [4]. Two systematic reviews have demonstrated a sex difference in the risk of developing schizophrenia [5,6]; both studies report that the male to female risk ratio is 1.4. Sex-specific associations with schizophrenia have previously been reported for a number of loci [7–11], but the robustness of these claims is open to doubt; results have yet to be corroborated [8–10] or replication has not been found with the same single nucleotide polymorphism (SNP) in the same direction in the same sex (e.g. [7,11]). This difficulty has afflicted attempts to establish sex-specific association in other diseases. An empirical assessment of 432 published sex differences in genetic association studies for different conditions found a single valid interaction that was consistently replicated in at least two other studies [12].

In the present study, we carried out a genome-wide association study using DNA pools of cases and controls constructed separately for men and women to allow the identification of sex-specific effects. Several studies have shown that DNA pooling detects the most promising loci with considerable savings in time and costs [13–18]. We previously scanned a three Mb region spanning the 22q11 microdeletion for association with schizophrenia using DNA pools. Our previous study [7,19] showed that the pools are representative of the allele frequencies in the sample and are adequate for detection of even modest association signals (odds ratios of about 1.3–1.5).

## Results

### DNA Pooling

Four separate pools of DNA were constructed as follows, 419 male cases, 241 female cases, 1,807 male controls and 964 female controls. Increasing the number of controls for a given number of cases significantly increases the power of the experiment. For example, our experiment with 660 cases and 2,771 controls is equivalent in power to about 1,100 cases and 1,100 controls [20]. Since estimating allele frequencies in pooled DNA samples introduces measurement errors, each of the four pools was independently analyzed in ten replicates with Affymetrix 500K SNP arrays. The results from the ten replicates were used to rank the SNPs (the 1,000 top rank-ordered SNPs are listed in Table S1).

**Figure 1:**
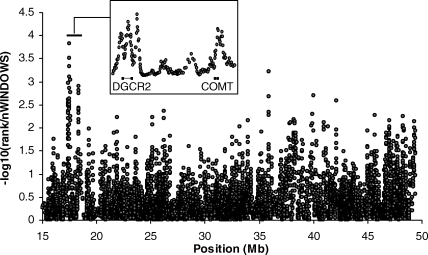
Association Signal on Chromosome 22 in a Sliding-window of Nine Consecutive SNPs The −log10 values are the negative logarithm (base 10) of the sliding windows ranks divided by the number of windows on Chromosome 22. The combined rank across men and women is based on a silhouette statistic [36]. The inset shows a magnification of the region indicated by a vertical line and the position of DGCR2 and COMT genes within this interval.

Our previously reported findings with several SNPs around both the COMT (116790) and DGCR2 (600594) genes in Chromosome 22 acted as a positive control for the performance of the current study. The association of multiple SNPs in the vicinity of both genes was clearly identified using our method (Figure 1). The best SNPs were selected for individual genotyping using an integrative approach taking into account their statistical significance, ranking and potential biological relevance (see Methods). As a result, a total of 194 SNPs were selected for individual genotyping.

### Individual Genotyping

The 194 selected SNPs were individually genotyped. At this stage, we used a sample of 759 controls and 745 patients from the Ashkenazi Jewish population. We genotyped a subset of the control sample used in the pools and enlarged the sample of cases (compared to the sample used in the pools), in order to differentiate in a cost-effective way between SNPs with real differences in allele frequencies between cases and controls and SNPs showing spurious differences due to technical anomalies in the DNA pooling procedure. Fifty-two SNPs out of 167 SNPs that passed our quality control (including a manual examination of genotype clusters and test of Hardy Weinberg equilibrium) showed *p*-values below 0.05 at any of the tests (male, female and combined) and nine SNPs had *p*-values below 0.005 (specific results for all SNPs are presented in Table S2).

The lowest *p*-value was found for SNP rs7341475 (G→A), for women only. SNP rs7341475 is located in Chromosome 7 (bp position = 103192051, NCBI Build 36), in the fourth intron of the *reelin* gene (RELN (600514)). This particular SNP was prioritized for individual genotyping because it resides within a gene previously studied for association with schizophrenia, in addition to having a high rank in the pools result (top 99.98% in the female pools). The common genotype of this SNP (GG) has a higher frequency in women with schizophrenia (75.5%) relative to female controls (59.3%) with an odds ratio (OR_GG_) of 2.1 (*p*_genotype_ = 9.8 × 10^−5^), and a significant gene-sex interaction (*p*_interaction_ = 5.3 × 10^−3^). In men, however, no effect was observed (OR_GG_ = 1.1, *p*_genotype_ = 0.47, GG frequency = 60.6%). Since the AA genotype is relatively rare, the genotype distribution in cases and controls was analyzed with the GA and AA genotypes grouped together. Similarly, the G allele was significantly overrepresented in women with schizophrenia (frequency = 86.6%) relative to female controls (frequency = 76.2%). The odds ratio for the G allele was OR_G_ = 2.0 with a corresponding allelic statistical significance of *p*_allele_ = 1.9 × 10^−5^. Again in men no effect was observed (OR*_G_* = 1.1, *p_allele_* = 0.38). The most significant result was obtained when the allele frequencies in female cases were compared to a combined sample of male and female control individuals (*p*_allele_ = 4.8 × 10^−7^). While a *p* < 5 × 10^−7^ cannot on its own be considered statistically significant under a stringent Bonferroni correction, it is suggestive evidence for true association under some assumptions [21], and therefore was further studied. We expanded the size of the Ashkenazi Jewish sample by increasing the number of controls (656 female and 1,988 males). We found increased evidence for the genotype association (*p*_genotype_ = 2.92 × 10^−5^; OR_GG_ = 2.0; Table 1) and the gene-sex interaction (*p*_interaction_ = 1.8 × 10^−4^). The association for males remained non-significant (*p*_genotype_ = 0.62).

**Table 1:**
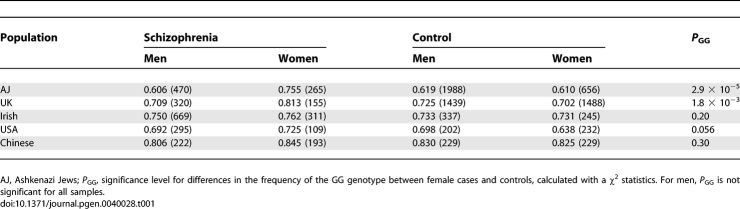
Frequency of GG Genotype (Sample Size)

We considered whether the result could be due to population stratification effect. We categorized parents of the Ashkenazi Jews individuals by their country of origin and compared the allele frequencies of rs7341475. We examined subjects whose parents were from the same country (77% of all individuals) and focused on those countries with more than 100 individuals (80% of individuals): Argentina, Germany, Russia, Poland, Ukraine, and USA (Figure S1). There was no significant difference in allele frequency between Ashkenazi Jewish individuals originated from different areas of the world (*p* = 0.9405).

**Figure 2:**
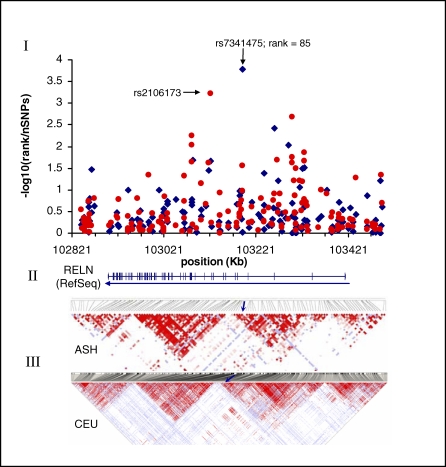
Association Signal for Schizophrenia in the Reelin Gene (I) The −log values are the negative logarithm (base 10) of the rank of each SNP divided by the number of SNPs on the array. The rank is based on a silhouette statistic [36], calculated separately for DNA pools of men (red) and women (blue) using the GenePool software. Arrows indicate the most significant SNPs, genotyped individually. The signal from rs2106173 was found to be an artifact of the DNA pooling experiment. (II) The structure and location of the reelin gene is from the Genome Browser of the University of California, Santa Cruz. Blue boxes represent the gene exons. (III) Similar patterns of linkage disequilibrium (LD) in Ashkenazi Jewish population (ASH) and individuals with European ancestry from HapMap (CEU). The blue arrows indicate the position of SNP rs7341475. Figures were generated using the Haploview software (http://www.broad.mit.edu/mpg/haploview/), with the standard color scheme. Pairwise LD levels between the SNPs in the region are represented by the color of the squares, which increase from white to blue to red (white, disequilibrium coefficient (D') < 1 and LOD score < 2; blue, D' = 1 and LOD score < 2; pink or light red, D' < 1 and LOD score ≥ 2; and bright red, D' = 1 and LOD score ≥ 2).

The association results and the linkage disequilibrium (LD) structure of RELN are presented in Figure 2. Based on HapMap LD data (CEU), there are no other SNPs in high LD (maximum *r* ^2^ = 0.4) with rs7341475 on the 500K Affymetrix array. One other SNP (rs2106173) in the RELN gene was also tested by individual genotyping but no statistically significant difference was found. The association signals for other SNPs in RELN (based on DNA pools) were moderate (maximum rank: for men = 1,062, for women = 1,969; Figure 2). We studied the patterns of LD in RELN in the Ashkenazi Jewish population using the genotypes of 129 SNPs (distributed between 102783336 and 103533335, NCBI Build 36) in 197 unrelated control individuals (obtained in the course of another study [22]). We found that the block structure in our sample from the Ashkenazi Jewish population is very similar to that of HapMap CEU. High correlations (*r* ^2^ > 0.8) are observed between rs7341475 and other SNPs distributed up to 39.8 kb upstream to rs7341475. The SNPs that are highly correlated with rs7341475 (rs10435342, rs6951931, rs17290575, rs6954479 and rs39327) are all located in the third or fourth intron of the gene. There is a substantial correlation (*r* ^2^ > 0.5) between rs7341475 and other SNPs in a 77.4 kb interval (103188857–103266305). There are no SNPs in neighboring genes in high correlation with rs7341475.

### Replication Study

**Figure 3:**
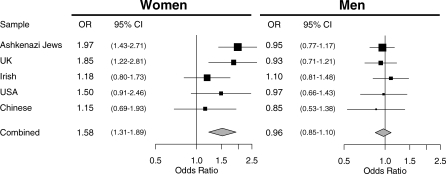
Risk Effects of the GG Genotype of rs7341475 Estimated by Odds Ratio Separately for Men and Women across Samples The point estimate of the odds ratio for each sample is represented by a square with a size proportional to the weight of study. The 95% confidence interval (95% CI) for each study is represented by a horizontal line. The confidence interval for the combined data is represented by a diamond.

To confirm the female-specific association between rs7341475 and schizophrenia, we tested rs7341475 in four other sample sets, three of European ancestry (UK, Ireland and USA) and one Chinese. In this replication study we tested a total of 2,274 cases (768 women and 1,506 men) and 4,401 controls (2,194 women and 2,207 men). We separated all samples by sex and tested the association for male and female subjects. Based on the association in the Ashkenazi Jewish population, in the replication samples we tested the prediction that the frequency of the GG genotype is increased in women with schizophrenia. The female-specific association of rs7341475 with schizophrenia was replicated in the UK case-control group with an effect in the same direction (O*R*_GG_ = 1.85; *p*_genotype_ = 1.8 × 10^−3^), and a significant sex effect (*p*_interaction_ = 3.2 × 10^−3^). All other populations showed an effect in the same direction, although individually the effects were not significant (Figure 3; Table 1). Combining all replication samples yielded a genotype OR_GG_ of 1.41 (95% CI = 1.13–1.76) with a corresponding *p*_genotype_ of 2.1 × 10^−3^ for women and OR_GG_ of 0.97 (95% CI = 0.83–1.15, *p*_genotype_ = 0.76) for men. The odds ratio for women in the combined replication set was significantly higher than men (*p*_interaction_ = 4.2 × 10^−3^). The association in the combined replication samples remained significant (*p*_genotype_ = 0.045) even after excluding the UK sample, which shows that the result of the meta-analysis is not attributable to a single highly significant sample.

The female specific OR_GG_, from all samples, including the initial Ashkenazi Jewish sample is 1.58 (1.31–1.89) with a corresponding *p*_genotype_ of 8.8 × 10^−7^, *p*_interaction_ of 1.6 × 10^−5^, and a female-population attributable risk of 50%. Note that the estimated risk effect including the Ashkenazi Jewish sample might be slightly inflated, because rs7341475 was selected as one of the best performing SNPs from a large number of SNPs examined. Thus an unbiased estimate for the risk of this SNP, should be based on the replication samples (OR_GG_ = 1.41, or 40% for the female attributable risk), even though there was no statistical evidence of heterogeneity of the odds ratios across the studies (*p* = 0.20). The frequency of the GG genotype, however, varies between populations (Table 1) – highest in the Chinese sample (frequency = 82.8%) and lowest in the Ashkenazi Jewish population (frequency = 61.6%).

## Discussion

We have carried out a genome-wide association analysis of schizophrenia, using pooled DNA. We identified one SNP in the fourth intron of RELN that confers a sex-specific risk of schizophrenia. Although the significance of the association between rs7341475 and schizophrenia in the Ashkenazi Jewish sample would not withstand a Bonferroni correction for multiple testing, we were able to replicate the sex-specific association in other samples from different populations that were tested for this specific SNP. The same allele and genotype are overrepresented in women, but not men, with schizophrenia in three different populations, Ashkenazi Jews, Europeans and Chinese (although the overrepresentation is not independently statistically significant in all populations tested). This observation, together with the fact that the association in the combined replication samples is significant and robust (even to the removal of the sample showing the most significant association) increases our confidence that we have found a genuine association.

The association observed in this study is unlikely to be the result of population stratification. The samples from the UK showing robust replication of the initial association were individually genotyped for SNPs across the whole genome. As such, this sample was rigorously evaluated for possible population stratification [21]. We also found no evidence for stratification in the Ashkenazi sample, indicating that the increase in allele frequency observed in women with schizophrenia cannot be caused by population structure in the Ashkenazi Jewish sample.

Genetic association studies have so far failed to report any consistent association between Reelin gene polymorphisms and schizophrenia [23–27]. However the gene has not so far been systematically screened. According to HapMap data, 183 tag SNPs would be needed to capture common variation at the gene (with *r* ^2^ > 0.8, MAF > 5%), while the SNPs on the Affymetrix 500K arrays capture 60% of SNPs. Most studies report data on a SNP in the promoter region, or on a CGG repeat polymorphism in the 5′-untranslated region; none have tested rs7341475. The two previously reported polymorphisms are located in LD blocks that do not contain rs7341475.

Our finding is important on two counts: first, it supports the hypothesis of a neurodevelopmental origin for schizophrenia, assuming that the genetic association reflects variation in the function of the RELN gene. Reelin, the protein product of RELN, is a serine protease [28] that acts via a number of receptor-mediated pathways on neurons [29]. It plays a key part in corticogenesis, as demonstrated by the cytoarchitectural abnormalities of the null mutant reeler (rl −/−) mouse [30]. RELN mutations in humans are associated with an autosomal recessive form of lissencephaly [31], a mental retardation syndrome that does not include psychosis.

Second, while a sex difference in the risk for schizophrenia has been found, its molecular basis has so far been unclear. Here we establish a replicated sex-specific association. Intriguingly, there is prior evidence for sexual dimorphism at the reelin locus. Sex effect have been noted in one study, reporting that RELN expression was higher in women compared to men (in layer I neurons) and a reduction in RELN expression observed only in men with schizophrenia (in the superficial interstitial white matter neurons). However, as the authors acknowledge, these have not been noted in other brain regions [32]. In mice, loss of Purkinje cell was observed in male mice with only one functional reelin gene (rl +/−) but not in reelin-deficient female mice [33].

Finally we note that sex hormones are likely to mediate changes in RELN expression: for example, administration of testosterone decreases reelin expression in the brains of male European starlings [34]. Our result of a female-specific association of RELN with schizophrenia may suggest a possible pathway where sex hormones modulate gene expression, which by altering cortical structure, increases susceptibility to psychosis.

## Materials and Methods

### Ashkenazi Jewish sample.

Samples from individuals diagnosed with schizophrenia were collected from hospitalized patients from seven medical centers in Israel (described in [7]). Schizophrenia was diagnosed by Diagnostic and Statistical Manual criteria (DSM-IV, American Psychiatric Association). Ashkenazi controls were collected from volunteers in blood banks. All four grandparents of each subject were of Ashkenazi Jewish origin, and all subjects (or the subject's legal representative) signed an informed-consent form.

### Other studied samples.

Chinese Han schizophrenia patients, their families and control subjects were recruited from Sichuan Province, SW China and consisted of 415 unrelated patients (222 males and 193 females) and 458 normal Han Chinese controls (229 males and 229 females). All patients were interviewed by an experienced psychiatrist using the SCID, and a diagnosis of schizophrenia was made according to DSM-III-R or DSM-IV criteria. Information was also collected from examination of medical records and all other available sources. Genomic DNA was extracted from peripheral blood according to standard phenol-chloroform methods. This study was approved by the South London and Maudsley Trust ethical committee and informed consent was obtained from all patients and control individuals.

The Irish Case – Control Study of Schizophrenia (ICCSS) samples were collected in Northern Ireland and the Republic of Ireland. Individual genotypes were obtained for 980 affected cases (669 males and 311 females) and 582 controls (337 males, 245 females). The affected subjects were selected from in-patient and outpatient psychiatric facilities in the Republic of Ireland and Northern Ireland. Subjects were eligible for inclusion if they had a diagnosis of schizophrenia or poor-outcome schizoaffective disorder by DSM-III-R criteria, which were confirmed by a blind expert diagnostic review. Excluding the relatively small number of schizoaffective female patients (*n* = 36) did not significantly alter the female-specific association result. Controls, selected from several sources, including blood donation centers, were included if they denied a lifetime history of schizophrenia. Both cases and controls were included only if they reported all four grandparents as being born in Ireland or the United Kingdom.

The UK sample consisted of unrelated subjects with schizophrenia (320 males and 155 females). All were white and born in the British Isles. All patients had a consensus diagnosis of schizophrenia according to DSMIV criteria made by two independent raters following a semi-structured interview by trained psychiatrists or psychologists using the Present State Examination or the Schedules for Clinical Assessment in Neuropsychiatry (SCAN) interview and review of case records. Cases were screened to exclude substance-induced psychotic disorder or psychosis due to a general medical condition. The mean age at first psychiatric contact was 23.6 (SD 7.7) years and the mean at ascertainment was 41.7 (SD 14.6) years. Multicentre and Local Research Ethics Committee approval were obtained, and all subjects gave written informed consent to participate. The control sample used by the Wellcome Trust Case Control Consortium (WTCCC) is described in detail elsewhere [21]. Briefly, controls (*n* = 2938) came from two sources, the 1958 British Birth Cohort (58C) and UK blood donors. At a genome wide level, the two groups do not significantly differ with respect to allele frequencies, justifying their use as a single control group. Individuals included in the study were living within England, Scotland and Wales. Individuals (*n* = 26) with non-Caucasian ancestry as determined by multidimensional scaling (MDS) were previously removed by the WTCCC from the sample. Controls were not screened for psychosis, but given the expected modest frequency in an unscreened sample, this has little effect on power. For the genome-wide association study from which these data were taken (manuscript submitted), λ was estimated at 1.06 genome-wide, which is at the lower end observed for other phenotypes when compared with the same controls. The call rate at this test locus for subjects in the genome-wide association study was >99.5% in each of cases and controls.

The USA sample consisted of unrelated subjects with schizophrenia (295 males and 105 females) and unrelated controls (202 males and 232 females) selected from a family-based sample that was ascertained as part of the Clinical Brain Disorders Branch/National Institute of Mental Health Sibling Study. All subjects were diagnosed using the Structured Clinical Interview (SCID). Probands met DSM-IV criteria for broad schizophrenia diagnosis. Control individuals were ascertained from the National Institutes of Health Normal Volunteer Office and were screened by SCID diagnosis for psychiatric disorders and excluded also if they had a first degree relative with a schizophrenia spectrum diagnosis. All participants gave informed consent and self-identified as Caucasian of European ancestry. All subjects also underwent extensive physical and laboratory screening to rule out complicating medical conditions and substance abuse.

### DNA pooling.

The DNA pools were constructed using DNA samples from 660 schizophrenic patients (419 males and 241 females) and 2,771 controls (1,807 males and 964 females). Pools were created by using equal aliquots of each sample, as described in ref. [7,19]. The pools were constructed using DNA samples from both sexes separately. Each pool (*n* = 4) was allelotyped ten times with the 500K Affymetrix arrays sets as previously described [18].

### SNP selection for individual genotyping.

We selected 194 unique SNPs using three different ranking methods (Figure S2), in addition to prioritizing SNPs in genes previously studied for association with schizophrenia (schizophrenia candidate genes). We used the SchizophreniaGene database [35] to identify genes that were previously tested for association with schizophrenia (515 genes were listed at the time of access [11/05/07]). SNPs with minor allele frequencies below 5%, according to HapMap (CEU) were excluded from further analysis due to the limited power to detect a significant association and the decrease in the accuracy of DNA pooling with rare variants.

Eighty SNPs were selected by the following criteria (some SNPs were selected by more than one method). We calculated for each SNP a modified chi-squared statistic, which includes the error variance introduced by the DNA pooling procedure [18,19] . SNPs were ranked based on logarithm (base 10) of the *p*-value (logP). Sixty first-ranked SNPs (30 from each array type, Nsp & Sty) were selected for individual genotyping. Additional twenty SNPs with logP > 3 were selected because they reside in candidate genes.

Fifty-five SNPs were selected by the following criteria. We used the GenePool software with silhouette statistic and Manhattan distance as the distance measure [36]. The SNPs were ranked based on the silhouette statistic separately for males and females patients, ranging from 1 for the highest silhouette score to 510,552 for the lowest score. We generated a combined rank for all samples based on the average rank in males and females weighted for the different size of samples. The first ten ranked SNPs with a minor allele frequency above 5% (based on HapMap, CEU) were selected for individual genotyping. This was done for each of three sets: male, female and combined. Twenty-five additional SNPs in candidate genes for schizophrenia were also selected for individual genotyping if they were also ranked in the first 1,000 SNPs in any of the sets.

Eighty-two SNPs were selected by the following criteria: first, we calculated the mean rank score in a sliding-window of nine consecutively neighboring SNPs. For each SNP we assigned a rank based on the mean score in the window showing the largest score out of the different possible windows. The sliding window method was used to identify regions where neighboring SNPs consistently show differences in allele frequency between cases and controls. This method minimizes spurious differences arising from technical anomalies in the analysis of pooled DNA [37], but could preferentially select SNPs in large blocks of LD. The analysis was carried out separately for male, females and the combined sets. SNPs in the first 1,000 ranked windows were sorted based on the SNP's individual rank score. The first 60 SNPs for the combined sample and the first ten for males and females samples were selected for individual genotyping. Two additional SNPs in candidate genes for schizophrenia that were also ranked in the first 1,000 SNPs in the combined analysis were also selected for individual genotyping.

### Individual genotyping.

DNA samples from the Ashkenazi Jewish and Chinese populations were genotyped using the Sequenom iPLEX system. LD in the Ashkenazi Jewish control sample was assessed using genotypes obtained from an Illumina HumanHap300 BeadChip in the course of another study [22]. Samples from the Irish Case – Control Study of Schizophrenia and from the US were genotyped using a Taqman 5′-exonuclease allelic discrimination assay. Quality checks for the Sequenom system included concordance rate (>98%) for genotypes of 83 DNA samples from HapMap cell lines, call rate (>95%), Hardy-Weinberg equilibrium in control (*p* > 0.001), manual checks of genotypes clusters and concordance rate (>98%) for re-genotyping. Genotypes for the UK sample were taken from a genome-wide association study performed in concert with the WTCCC study of common diseases [21] based upon the GeneChip 500K Mapping Array Set. Samples were genotyped at the Affymetrix service laboratory in San Francisco (USA) using the same pipeline as the WTCCC disease and controls samples.

### Statistical analysis.

All data analysis was performed using the R language and environment for statistical computing (http://www.r-project.org/). Single-SNP analysis for the individual genotyping data was carried out using a χ^2^ test on allele and genotype counts. For the replication samples a one-tailed test was employed, since we tested a specific hypothesis of an increase in GG frequency in female patients and not other possible directions of association. We combined the rare homozygote genotype with the heterozygotes for the genotype association analysis. Hardy-Weinberg was assessed using the χ^2^ statistic with one degree of freedom. To test for a gene by sex interaction, a z-score was calculated using a ratio between the difference in the natural logarithm of the odds ratio between males and females and the square root of the variance of the difference. We used the Mantel-Haenszel method to combine the data of different populations with a fixed effect model and Cochran's Q statistic to test for heterogeneity as implemented in the R package ‘meta' (version 0.5). LD was calculated using the Haploview software package. Population attributable risk of rs7341475 was calculated for women as (*K* − 1)/*K,* where *K* = ∑*f_i_* × *g_i_; f_i_* is the frequency of the *i* genotype, and *g_i_* is the estimated genotype relative risk of the *i* genotype assuming multiplicative model.

## Supporting Information

Figure S1 Allele Frequency of rs7341475 in Ashkenazi Jews from Different Origin The height of each bar represents the frequency of the G allele of rs7341475 in Ashkenazi Jews originated from different countries. Error bars are 95% confidence interval for the frequency. The vertical solid line is the frequency of the G allele in schizophrenia female patients and the dashed lines are the 95% confidence interval for that frequency.
(42 KB DOC)

Figure S2 SNP Selection for Individual Genotyping The diagram shows the method used to select SNPs for individual genotyping. SNPs were selected based on three ranking methods. SNPs were also prioritized in each ranking system if they reside within genes previously studied for association with schizophrenia (see text for more details).
(28 KB DOC)

Table S1 Results of DNA Pooling The 1,000 top rank-ordered SNPs based on a silhouette statistic calculated using the GenePool software for men, women, and combined sample.
(321 KB XLS)

Table S2 Results of Individual Genotyping (52 KB XLS)

### Accession Numbers

The Online Mendelian Inheritance in Man (OMIM) (http://www.ncbi.nlm.nih.gov/Omim/) accession numbers for disease and genes mentioned in the paper are Schizophrenia (181500), RELN (600514), COMT (116790), and DGCR2 (600594).

## Abbreviations

CEU: Utah residents with ancestry from northern and western Europe
LD: linkage disequilibrium
MAF: minor allele frequency
OR: odds ratio
SNP: single nucleotide polymorphism
