# Supplementary material for: Genome-Wide Association Identifies a Common Variant in the Reelin Gene That Increases the Risk of Schizophrenia Only in Women
Source: PLoS Genet. 2008 Feb 15;4(2):e28. doi: 10.1371/journal.pgen.0040028 (PMC2242812; doi:10.1371/journal.pgen.0040028)
Supplement: Supplementary file 1 [file pgen.0040028.sg001.doc]

**Supplementary Figure S1.**The height of each bar represents the frequency of the G allele of rs7341475 in Ashkenazi Jews originated from different countries. Error bars are 95% confidence interval for the frequency. The vertical solid line is the frequency of the G allele in schizophrenia female patients and the dashed lines are the 95% confidence interval for that frequency.
