# Supplementary material for: Genome-Wide Association Identifies a Common Variant in the Reelin Gene That Increases the Risk of Schizophrenia Only in Women
Source: PLoS Genet. 2008 Feb 15;4(2):e28. doi: 10.1371/journal.pgen.0040028 (PMC2242812; doi:10.1371/journal.pgen.0040028)
Supplement: Supplementary file 2 [file pgen.0040028.sg002.doc]

**Supplementary Figure S2.** The diagram shows the method used to select SNPs for individual genotyping. SNPs were selected based on three ranking methods. SNPs were also prioritized in each ranking system if they reside within genes previously studied for association with schizophrenia (see text for more details).
